# Accuracy improvement in financial sanction screening: is natural language processing the solution?

**DOI:** 10.3389/frai.2024.1374323

**Published:** 2024-11-22

**Authors:** Seihee Kim, ShengYun Yang

**Affiliations:** ^1^Hana Bank, Seoul, Republic of Korea; ^2^Research Centre Business Innovation, Rotterdam University of Applied Sciences, Rotterdam, Netherlands

**Keywords:** financial institutions, financial sanction screening, fuzzy matching algorithm, Natural Language Processing (NLP), sensitivity, text similarity

## Abstract

Sanction screening is a crucial banking compliance process that protects financial institutions from inadvertently engaging with internationally sanctioned individuals or organizations. Given the severe consequences, including financial crime risks and potential loss of banking licenses, effective execution is essential. One of the major challenges in this process is balancing the high rate of false positives, which exceed 90% and lead to inefficiencies due to increased human oversight, with the more critical issue of false negatives, which pose severe regulatory and financial risks by allowing sanctioned entities to go undetected. This study explores the use of Natural Language Processing (NLP) to enhance the accuracy of sanction screening, with a particular focus on reducing false negatives. Using an experimental approach, we evaluated a prototype NLP program on a dataset of sanctioned entities and transactions, assessing its performance in minimising false negatives and understanding its effect on false positives. Our findings demonstrate that while NLP significantly improves sensitivity by detecting more true positives, it also increases false positives, resulting in a trade-off between improved detection and reduced overall accuracy. Given the heightened risks associated with false negatives, this research emphasizes the importance of prioritizing their reduction. The study provides practical insights into how NLP can enhance sanction screening, while recognizing the need for ongoing adaptation to the dynamic nature of the field.

## 1 Introduction

Sanction screening is a crucial process for identifying, preventing, and mitigating sanction risks in the banking sector (The Wolfsberg Group, 2019). Financial institutions use sanction screening programs to verify all parties involved in their banking activities against sanction lists. Compliance with sanction regulations, which involves refraining from processing transactions or opening bank accounts for sanctioned entities, is a mandatory requirement for financial institutions. Failure to adhere to these rules can lead to severe consequences, such as financial crime implications, hefty fines, and potential threats to the institution’s banking license. For instance, ING faced a €775 million fine in 2018 for money laundering and terrorism financing failures, while ABN AMRO paid €480 million in 2021 for maintaining relationships with suspicious clients (Sterling and Meijer, 2018; Deutsch and Meijer, 2021). Given these risks, banks and risk solution providers are keen on implementing highly accurate sanction screening programs.

Sanction screening is a key area that has undergone digitalization within the banking industry. Traditionally, the vetting of suspicious clients and transactions was done manually. However, with global banks processing more than 200,000 transaction requests daily from their customers (ING, 2022), and an annual customer increase of 500,000 (ING, 2020; ING, 2021), manual scrutiny of every transaction and customer is impractical. Manual screening also poses operational risks due to potential human errors and slow processing speeds. Consequently, financial institutions have adopted sanction screening software to identify suspicious transactions. Nonetheless, these programs are not infallible and cannot guarantee 100% accuracy.

The accuracy of a sanction screening program is contingent on the specific rules and thresholds set by each bank. Different countries have distinct regulations and internal policies for monitoring customers and transactions, making program design more complex with increased requirements. Poorly designed programs may result in low output accuracy. Selecting the alert generation threshold is also crucial; a low threshold, such as 70% similarity, can lead to numerous false positives, while a high threshold, like 99%, may reduce false positives but raise the risk of false negatives.

The proliferation of sanctions measures since the 1950s, with a significant upsurge in the 2010s, underscores the need for more sophisticated and complex screening algorithms (Felbermayr et al., 2020; Van Bergeijk, 2022). The primary algorithm for text-similarity checks in sanction screening is the Levenshtein distance algorithm, which compares inputted names with those on sanctions lists. Sanction screening programs employ “fuzzy matching,” considering local languages, pronunciation, spelling, and abbreviations (The Wolfsberg Group, 2019). These programs also incorporate various matching rules to generate alerts while accounting for the tolerance of inputted names.

Table 1 outlines the potential outcomes and risks associated with sanction screening. True positives and true negatives are not a concern, as the program correctly predicts the identity of the screening target. The issues lie with false negatives and false positives. False negatives are alerts that are missed against a target predicted as non-sanctioned but is, in fact, sanctioned. This is the least expected and most critical situation, leading to severe consequences in onboarding or transactions with sanctioned entities. A false negative necessitates an immediate algorithm revision.

**Table 1:** Sanction screening program risk matrix.

Real			
Prediction		*True*	*False*
	*Positive*	True positive: successful to detect the sanctioned entity	False positive: not dangerous, but lowers efficiency and accuracy - > need improvement
	*Negative*	False negative: disastrous, the program fails to detect the sanctioned entity - > needs immediate improvement	True negative: fine, since the program does not alert the non-sanctioned entity

The other issue is false positives, which are fake alerts against non-sanctioned targets that the program incorrectly deems to match sanctioned parties. While less severe than false negatives, high false positive rates necessitate human intervention to verify accuracy, leading to inefficiencies and an increased risk of human error.

Despite the use of various algorithms, the current sanction screening program’s accuracy remains poor, with false positive hits comprising over 90% of all alerts. Financial experts are exploring advanced technologies, particularly artificial intelligence (AI), as a solution to this limitation (PwC, 2019; Goethals and Decraemere, 2020).

AI, particularly in the form of Natural Language Processing (NLP) and text mining techniques, holds promise for improving screening controls and reducing operational costs. NLP combines human language with machine learning models, enhancing the recognition of named entities and resolving word ambiguities in text similarity checks.

This study aims to answer the research question: How does the adoption of NLP improve the accuracy of the sanction screening program? Our experiment found that NLP, when applied to fuzzy matching weights, enhances the accuracy of text similarity checks. It outperforms the version without NLP in eliminating false negatives and detecting true positives at a specific fuzzy matching threshold. NLP distinguishes between names of individuals and organizations, contributing to rational and efficient fuzzy weight schemes by utilizing input data.

While NLP significantly reduces false negatives, it also generates more false positive alerts due to its conservative approach to sanctioned name matching. This trade-off between general accuracy and sensitivity to suspicious cases complicates program performance evaluation. Therefore, prioritizing firm requirements is crucial to guide program development and achieve its primary goal.

This paper follows a structured approach. The “Theoretical Background” section provides an overview of established technologies and techniques within the sanction screening program. Subsequently, the “Research Method” section outlines the research methodology, encompassing experiment design and variable measurement. The “Research Findings” section offers an in-depth examination of the experiment’s outcomes. Following this, the “Discussion” section delves into an extensive exploration of the results. Finally, the “Conclusion” section encapsulates the paper with a concise summary of findings, emphasizing both academic and practical contributions.

## 2 Theoretical background

The sanction screening program employs text similarity check technologies to match customer lists with sanctions lists and generate alerts when matches are found. These technologies are rooted in text similarity methods, widely used in information management tasks like text classification and information retrieval (Gomaa and Fahmy, 2013). The program currently relies on fuzzy matching techniques, and in this section, we will review these techniques and explore the potential adoption of NLP.

### 2.1 Fuzzy matching

The current sanction screening program employs various fuzzy matching techniques to compare and filter names from the sanctions list when a user checks a customer’s name. Fuzzy matching algorithms produce results that are identical or reasonably close matches by using a similarity function (Chaudhuri et al., 2003). This approach acknowledges that natural language is not easily translated into binary (0 or 1) due to its inherent uncertainties, vagueness, and imprecision, encompassing personal sentiments, tone, and emotions. Fuzzy matching adapts to these characteristics (Gupta et al., 2018).

Several fuzzy matching techniques have been developed, each with distinct advantages and disadvantages, offering efficient solutions in descriptive and predictive data analytics (Gupta et al., 2018). Lieu (2012) examines five different fuzzy matching techniques: edit distance, common key, list, statistical similarity, and word embedding methods, outlining their pros and cons. Table 2 summarizes Lieu’s findings and additionally evaluates the strengths and weaknesses of NLP as a potential companion to fuzzy matching.

**Table 2:** Advantages and limitations of fuzzy matching techniques (Adapted from Lieu, 2012) and NLP.

Techniques	Examples	Advantages	Limitations
Edit distance method	Levenshtein distance Jaro-Winkler distance Jaccard similarity coefficient	Easy to implement Widely used	Limited to Latin-based languages All swaps are weighted evenly Missing linguistic nuances
Common key method	Soundex Metaphone Double Metaphone Beider-Morse Phonetic Matching Caverphone	Fast execution High recall	Mostly limited to Latin-based languages (transliterating non-Latin names reduces precision)
List method		Easy to maintain	Computationally intensive Less flexible, cannot handle unexpected variations Heavy to process Slower performance
Statistical similarity method		Matches across languages and scripts High accuracy	Slower performance Higher barrier to entry, requiring significant training data
Word embedding method		Makes semantic matches that a spelling centric method would miss	Only relevant to organization name matching
Natural language processing	Text classification Text extraction Machine translation Natural language generation Sentiment analysis	Improved efficiency of documentation Named entity recognition Combination of linguistics and statistical methods	Poor performance with the imprecision and ambiguity in human language Cannot catch evolving use of language

Each technique has its unique merits and drawbacks, making the choice of technology contingent on a clear understanding of each one’s logic. Notably, Levenshtein distance, as part of the edit distance method, is the most commonly used technique in the sanction screening program (Nino et al., 2019).

The list method preserves all possible variations of spelling outcomes, offering ease of management by adding or removing data. However, it demands significant data storage and can slow down text matching due to its heavy database.

The statistical similarity method trains models with thousands of name pairs to identify similar name pair features, ensuring high accuracy but slower execution and a higher barrier to adoption.

The word embedding method leverages semantic meaning to match names, allowing even entirely different words with similar meanings to be considered as matches. This technique has limitations, primarily applicable to organization name matching and unsuitable for proper nouns due to its vocabulary limitations.

Fuzzy matching’s limitation is its limited or lack of linguistic perception in detecting word similarities (Vanallemeersch and Vandeghinste, 2014). Considering the challenges posed by large, heterogeneous, and qualitative datasets, mixed data structures, and data uncertainty, NLP stands out as a technology to enhance and complement the inherent weaknesses of fuzzy matching. NLP leverages linguistic and statistical computation for text analysis, offering the potential to improve the accuracy of sanction screening results.

#### 2.1.1 Levenshtein distance

Levenshtein distance is a commonly used algorithm for text matching (Vanallemeersch and Vandeghinste, 2014; Nino et al., 2019). It falls under the edit distance methods and is appreciated for its ease of implementation. This algorithm calculates the minimal number of corrections, which include insertions, deletions, or substitutions, required to match two different words (Levenshtein, 1966). The core of the algorithm involves a sequential comparison of each segment in two different words.

**Figure 1:**
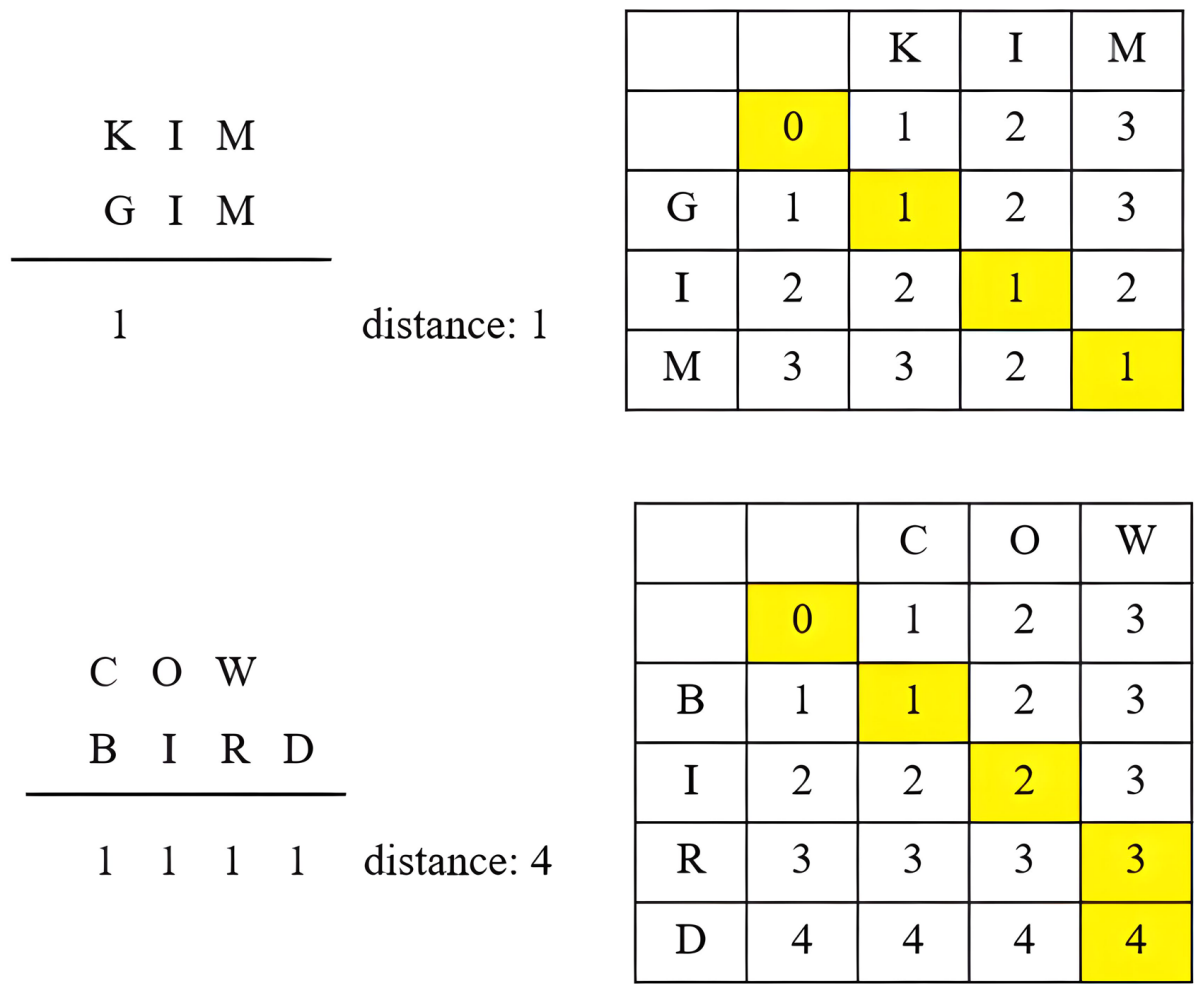
Phone string comparison in Levenshtein distance (adapted from Kruskal, 1983).

Levenshtein distance has found applications in dialect distance research, effectively measuring phonetic distances (Heeringa, 2004). Kessler (1995) employs four approaches with Levenshtein distance: phone string comparison, feature string comparison, all-word approach, and same-word approach. Phone string comparison is the simplest, treating all strings as equal units, with a distance of 1 for each substitution. For example, the distance between “Kim” and “Gim” is 1 due to the substitution of [K] and [G]. The distance between “Cow” and “Bird” is 4, accounting for substitutions of [C]/[B], [o]/[i], [w]/[r], and the addition of [d]. Figure 1 illustrates the distance calculation using the phone string comparison approach.

While this method is easy to apply, it struggles to differentiate between small and large phonetic differences. For instance, [b] and [f] sounds are treated equally, even though they may be more similar than [a] and [f] sounds. The feature string comparison approach provides more accurate phonetic distance measurements by pairing similar phones. However, this approach is complex as it necessitates analysis of articulation organ positions based on phonological knowledge. Kessler (1995) also considers the linguistic meaning of words when using the phone string comparison, termed the all-word approach. The same-word approach indicates that Levenshtein distance is employed only if two words are phonetically similar and have different meanings.

An advanced version of Levenshtein distance, known as Levenshtein automata of degree n, lists similar words when the Levenshtein distance between the input word and other words does not exceed “n” (Schulz and Mihov, 2002). This facilitates quick spelling error correction and suggests alternative words within datasets featuring similar spellings.

#### 2.1.2 Fuzzy matching weighting

Fuzzy matching offers enhanced text similarity checking by considering prior factors, making it an effective name matching technique. For sanctioned individuals, four key data types are typically provided by the sanctioning bodies: “name,” “date of birth (DOB),” “place of birth (POB),” and “nationality.”

Each bank must determine optimal weightings for building a Risk Score Card, tailoring the fuzzy matching process to its customer data history. Compliance and risk personnel assign weights to each data type to improve matching quality within the sanction screening program. For example, Nino et al. (2019) allocate 50% weight to “Full Name,” 20% to “Birth Year,” and 30% to “Associated Country” to create a Fuzzy Risk Score Assessment Framework. In the Netherlands, a common practice assigns 70% weighting to “name” and 15% each to “DOB” and “POB” because these weights are efficient for customer screening.

The Levenshtein distance algorithm operates effectively under these weight allocations, with each element having its own scoring criterion. Name matching yields the score of fuzzy matching, while DOB and POB require a 100% match. This approach is logical because sanctioned entity names can vary significantly, including nicknames, abbreviated names, and spelling variations, especially when not originating from a Latin-based language. Benchmark sanctions list data may not precisely match the name, but there remains a possibility that the individual associated with the name is the sanctioned party. To align with the sanction screening program’s primary goal of generating alerts with a high level of suspicion, establishing a threshold solely based on 100% exact name matching carries the potential risk of elevating the rate of false negatives. Hence, it is imperative that identity verification extends beyond mere name comparisons. Comprehensive verification should involve cross-referencing not only names but also DOB and POB at a 100% match to ascertain whether the identified entity is genuinely the sanctioned individual or entity.

Table 3 illustrates how the weighting mechanism of fuzzy matching functions in the sanction screening program. The program calculates Levenshtein distance between input data and the benchmark sanctions list dataset, assigning higher scores for more accurate matches. Users can set the program to generate alerts based on total matching scores, allowing for flexibility depending on the specific use case encountered by banks in practice. Testing the targeted dataset and determining the optimal threshold is essential to minimize false negatives while keeping false positives low.

**Table 3:** Weighted scorecard mechanism in sanction screening program.

	Name (a)	DOB (b)	POB (c)	Summed score (a + b + c)
Weights	70 points	15 points	15 points	100 points
Alert generation threshold	80%	100%	100%	
Case 1: If an input has 80% name match/ 100% DOB match/ 100% POB match	70 points × 80% = 56 points	15 points × 100% = 15 points	15 points × 100% = 15 points	86 points
Case 2: If an input has 90% name match/ 0% DOB match/ 0% POB match	70 points × 90% = 63 points	15 points × 0% = 0 points	15 points × 0% = 0 points	63 points
Case 3: If an input has 75% name match/ 100% DOB match/ 100% POB match	70 points × 75% = 52.5 points	15 points × 100% = 15 points	15 points × 100% = 15 points	82.5 points

### 2.2 Natural Language Processing

NLP is the technology for computer understanding and manipulation of human language in text or speech (Chowdhury, 2003). It encompasses machine learning processes drawing from computer science, AI, linguistics, psychology, mathematics, and information science. The necessity for NLP arises from the need to navigate the inherent ambiguity of natural language in the digital world.

NLP’s comprehension of natural language begins at the word level, progresses to sentence-level analysis involving word order, grammar, and sentence meaning, and ultimately encompasses the context of the entire document, acknowledging the variability of word and sentence meaning within different contexts.

**Figure 2:**
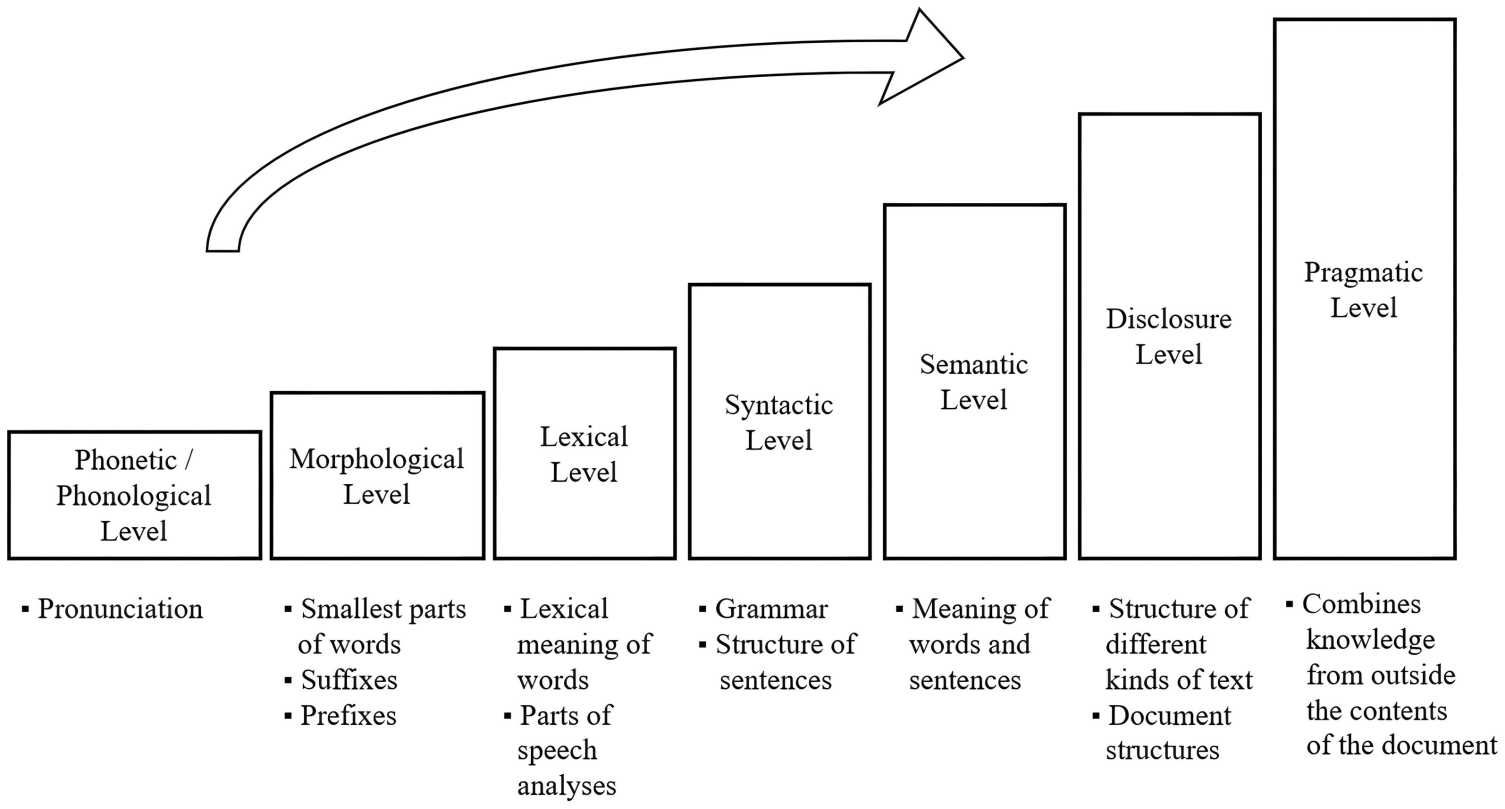
Comprehension progress of natural language (adapted from Chowdhury, 2003).

Comprising seven interdependent levels, the comprehension process intensifies as it progresses: phonetic/phonological, morphological, lexical, syntactic, semantic, discourse, and pragmatic levels (Chowdhury, 2003). Each level targets more advanced language aspects, offering multiple points of application for NLP (see Figure 2).

At the morphological level, NLP initiates data tokenization, dividing input strings into tokens as the first step in data pre-processing (Grefenstette, 1999). Tokenization facilitates the computer’s basic processing of text data by interpreting and grouping tokens into higher-level units. An example of basic tokenization is white space tokenization, dividing strings at blank spaces and grouping identical chunks for further analysis and model development.

NLP has made inroads in the financial domain, particularly in Financial Technology (FinTech) and Regulatory Technologies (RegTech). Applications include AI chatbots for conversational banking, text summarization algorithms, voice-based banking services, and RegTech solutions for efficient banking compliance management.

While NLP has not been widely adopted in current sanction screening practices due to the associated risks (Chartis Research Staff, 2019), its potential to address limitations in the existing program is worth exploring. The current sanction screening program faces challenges in distinguishing diverse pronunciation expressions and handling non-Latin alphabet origin names and names with prefixes or suffixes from different language backgrounds. NLP can potentially address these issues by adopting phonetic and morphological level analysis.

Furthermore, by adopting NLP at the lexical level, the program could differentiate between the meanings of commonly used terms. This differentiation is particularly relevant when sanctioning organizations with names that include terms not typically used in personal names, leading to false positives in the current system.

Overall, NLP has the potential to optimize fuzzy matching weightings for the program, improving the accuracy of name match predictions. This, in turn, could enhance the overall accuracy of sanction screening results, particularly in reducing false positives.

## 3 Methodology

In this section, we outline our methodology for examining the impact of NLP on the performance of sanction screening programs. Our approach was experimental, involving data collection from sanctions lists and the development of a demonstration version of the sanction screening program. We compared two scenarios: the As-Is version and the To-Be version, which included NLP. The goal was to demonstrate how NLP can enhance the effectiveness of sanction screening programs.

### 3.1 Experimental requirements

To develop a demonstration version of the sanction screening program, the initial step involved identifying essential requirements. These requirements served as the foundation for shaping the program’s functionality and constraints. The primary factors to consider when defining the program’s requirements included target, context, frequency, rules, and risk management (The Wolfsberg Group, 2019).

Furthermore, it is crucial to distinguish between two categories of requirements: functional requirements, which specify what the sanction screening program must achieve, and non-functional requirements, which delineate specific, quantified limitations the program must adhere to. Additionally, alongside the sanction screening program, data management must be integrated into the requirement identification process, as detailed in Table 4, summarizing both functional and non-functional requirements.

**Table 4:** Requirements of the sanction screening program implementation.

	Functional requirements (the system must be able to perform)	Non-functional requirements (quantified constraints or restrictions)
Sanction screening program	The program must detect and generate alerts for the true positives with a possibility of 100%. The program must judge the alert generation based on the sanction lists. The text similarity check function embedded in the program must perform well.	False positive rates shall be lower than 95%. Methods: adopting fuzzy matching technique
Data management	The sanction list data set must be updated as a latest version. The data which is no longer risk-relevant must be deleted immediately.	Data monitoring and data repairment shall be processed daily.

### 3.2 Experiment design matrix

The development of a demonstration version of the sanction screening program in this research used Python. Python proves to be an apt programming language for implementing the Levenshtein distance and NLP, which are pivotal components of this research. However, it is important to acknowledge that building a demonstration version has limitations; it cannot replicate all the features of the comprehensive sanction screening program developed by professional software engineers in the industry. Their program is a sophisticated integration of various sanction screening techniques devised by banking compliance experts.

Nonetheless, the fundamental technology underlying the sanction screening program involved the implementation of the Levenshtein distance algorithm for name matching. This core aspect justified our academic pursuit. It is vital to clarify that the demonstration version used in this experiment was a simplified representation of the actual sanction screening program. This demonstration version incorporated the Levenshtein distance algorithm with default fuzzy matching weights to perform text similarity checks.

The experiment unfolded in two distinct phases:

Phase 1: As-Is version of the sanction screening program.

The first phase encompassed the current state of the sanction screening program, based on the Levenshtein distance algorithm with default fuzzy matching weights. In practical sanction screening applications, human users typically play a pivotal role in assigning weights to different data types. The As-Is model in this first phase adhered to a foundational weighting scheme, mirroring the standard practice within the industry. As mentioned in Section 2.1.2, this customary weighting distribution often allocates 70% of the weight to the name, while the DOB and POB receive 15% each.

Phase 2: To-Be version of the sanction screening program.

The second phase explored the potential To-Be version of the sanction screening program. In this phase, the To-Be version employed the Levenshtein distance algorithm as the foundational text matching component, striving for algorithmic consistency. Additionally, it introduced the Cosine similarity algorithm as an alternative logic for text similarity checks, in an effort to enhance matching through NLP.

Phase 2 was designed to integrate NLP techniques to refine the fuzzy matching weighting scheme. While fuzzy matching weights are commonly assigned based on expert and practitioner experience in banking compliance, this arbitrary allocation may not always be optimal for all input data. NLP offers a solution for objectively determining rational weights for each data type by analyzing the entire input dataset and discerning which elements significantly influence more accurate text matching.

The primary objective of Phase 2 was to investigate whether the adoption of NLP for fuzzy matching weights leads to improved accuracy in text similarity checks. To evaluate this, the results obtained from the Phase 2 To-Be version will be measured and compared with those from the As-Is version of Phase 1.

### 3.3 Treatment design

The treatment that distinguished the As-Is human-devised version from the To-Be NLP-devised version revolved around fuzzy matching weights. These weights varied based on the type of customers. Both in the mock-up test set and sanctions lists, individual entities encompassed four distinct attributes that contribute to specifying the entity. In this context, the name held the highest importance, accounting for 70% of the overall weight in name matching. The remaining attributes, DOB, City of Birth, and Country of Birth, carried weights of 15, 7.5, and 7.5%, respectively. In contrast, for organization entities, the name retained 70% of the total importance, while the Country of Operation took up the remaining 30%. These settings, 70-15-15 (divided into 7.5–7.5) for individuals and 70–30 for organizations, form the fundamental configuration for fuzzy matching weights, aligning with the prevailing approach in current sanction screening practices. The specifics of the weight allocations were adjusted and their relevance validated through a pilot experiment.

The initial approach for setting default weights for the As-Is text similarity check program, based on the Levenshtein distance algorithm, comprised assigning weights of 70% for individual entities and 30% for organizations. To ensure the performance of the demo sanction screening program before the main experiment, a pilot version of the experiment was conducted.

A subset of 350 data points, randomly selected from the mock-up dataset, constituting 70% of the entire test set, was used to assess the impact of the adjustments. The program was configured to trigger an alert for cases where the fuzzy matching score of the text match exceeded 80.

**Figure 3:**
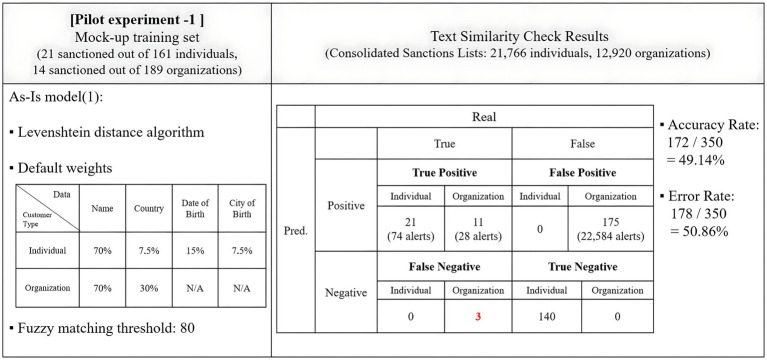
Results of the pilot experiment.

The pilot experiment adhered to the default weighting scheme and assessed if it aligned with the current standards in sanction screening programs. It was expected to achieve an accuracy rate of over 25%, and the demonstration version recorded an impressive 49.14% accuracy, satisfying this criterion. The results of the pilot experiment were summarized in Figure 3.

The program demonstrated success in filtering out all 21 sanctioned individuals in the mock-up training set while generating 74 cases of alerts. Remarkably, it did not produce any false positive alerts, contributing to a higher accuracy rate. However, the program performed suboptimally in screening organizations. It missed three cases of true positives, categorized as three false negatives. Furthermore, it generated 175 false positives, with 22,584 alerts, suggesting that it wrongly categorized all true negatives as positives.

To enhance the accuracy of screening organizations, adjustments were made to the weight allocation for each data type, changing from 70–30 to 80–20 for the name and country. The initial fuzzy matching threshold for hit generation, set at 80, was adjusted to 85 to minimize the number of false positives. These adjustments aimed to determine if the NLP adoption for fuzzy matching weights led to an improvement in the accuracy of text similarity checks.

**Figure 4:**
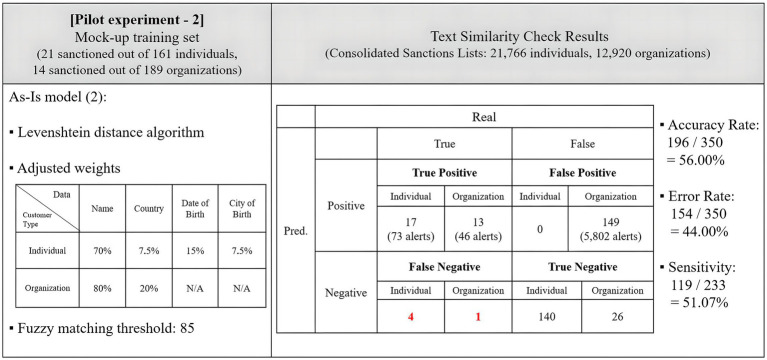
Results of the pilot experiment with adjustments.

The adjustments to the fuzzy matching schemes effectively reduced the number of false positives and associated alerts. A second round of testing reflected these adjustments, with the main findings summarized in Figure 4.

Under these adjustments, the program saw an increase in the number of false negatives, rising from 3 to 5. However, the number of false positives decreased from 175 to 149. The accuracy rate increased to 56.00%, with the program making 196 correct predictions out of 350 trials. This time, the sensitivity rate was also calculated. Within the sanctions lists, there were 233 correct matches that corresponded to the 50 sanctioned entities in the mock-up data, considering official names and aliases. The program detected 119 matches, leading to a sensitivity rate of 51.07%. The concept of sensitivity will be explored further in Sections 3.4.

The next aspect to investigate in the pilot study was the selection of an appropriate algorithm for integrating NLP. This research applied NLP to the Levenshtein distance algorithm as the base program to evaluate whether NLP can offer an effective solution for current sanction screening programs.

Each model was subjected to testing using the same parameters as those employed in the As-Is version. An identical dataset comprising 350 data points was used to evaluate the program, with the fuzzy matching threshold set at 85. NLP dynamically configured the weighting scheme through its machine learning process. The code execution time for running the second model was recorded at 3 h and 8 min.

**Figure 5:**
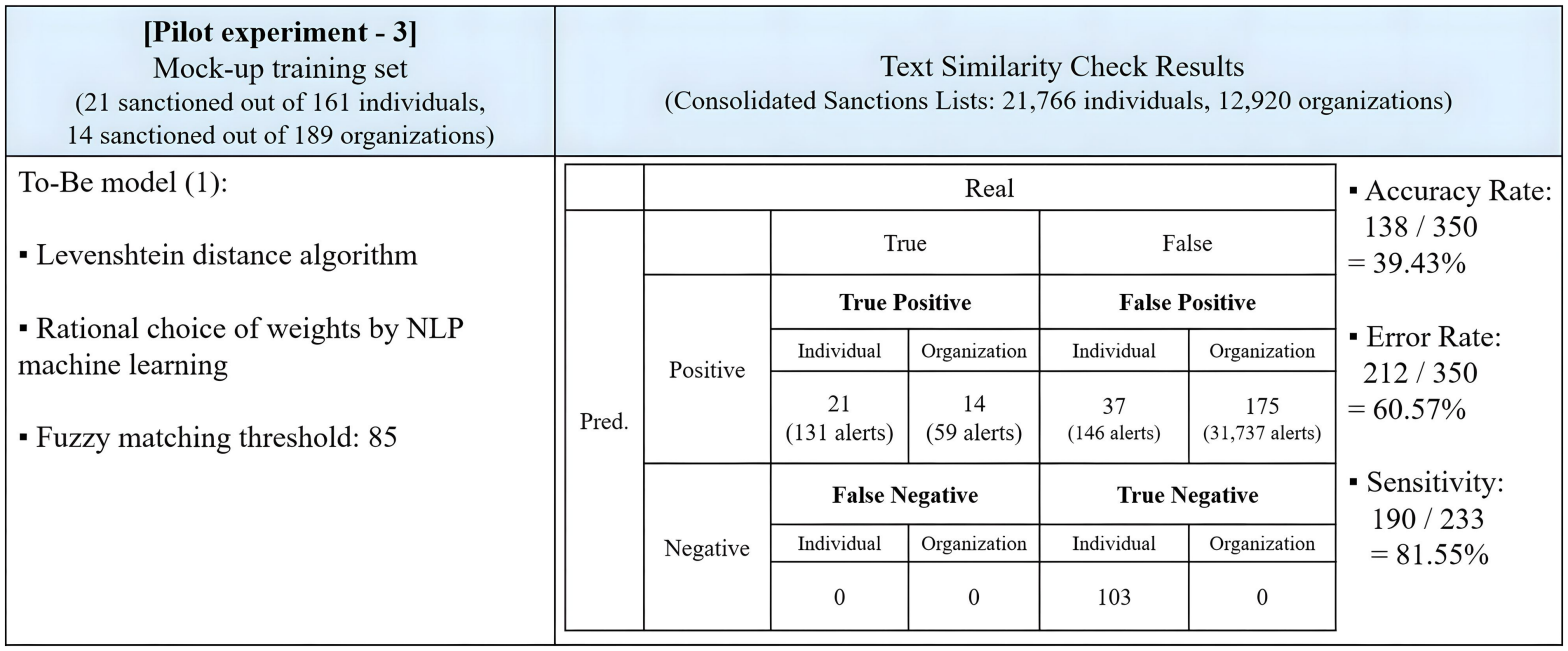
Results of the pilot experiment of NLP adoption on the Levenshtein distance algorithm.

Figure 5 displays the results of this pilot experiment. The program demonstrated improved performance in capturing all 35 true positive entities, achieving zero false negatives, surpassing the As-Is version. However, its accuracy rate decreased to 39.43%, resulting in 63 false positives more than the As-Is model. The program generated more alerts for both individuals and organizations. Notably, it categorized all true negative organizations as positives, producing 31,737 false positive alerts. Compared to the As-Is version of the Levenshtein name matching program, the To-Be version exceled in identifying true positive matches but fell short in reducing false positives. The sensitivity rate was 81.55%, marking a 30.48%-point improvement compared to the As-Is model.

**Figure 6:**
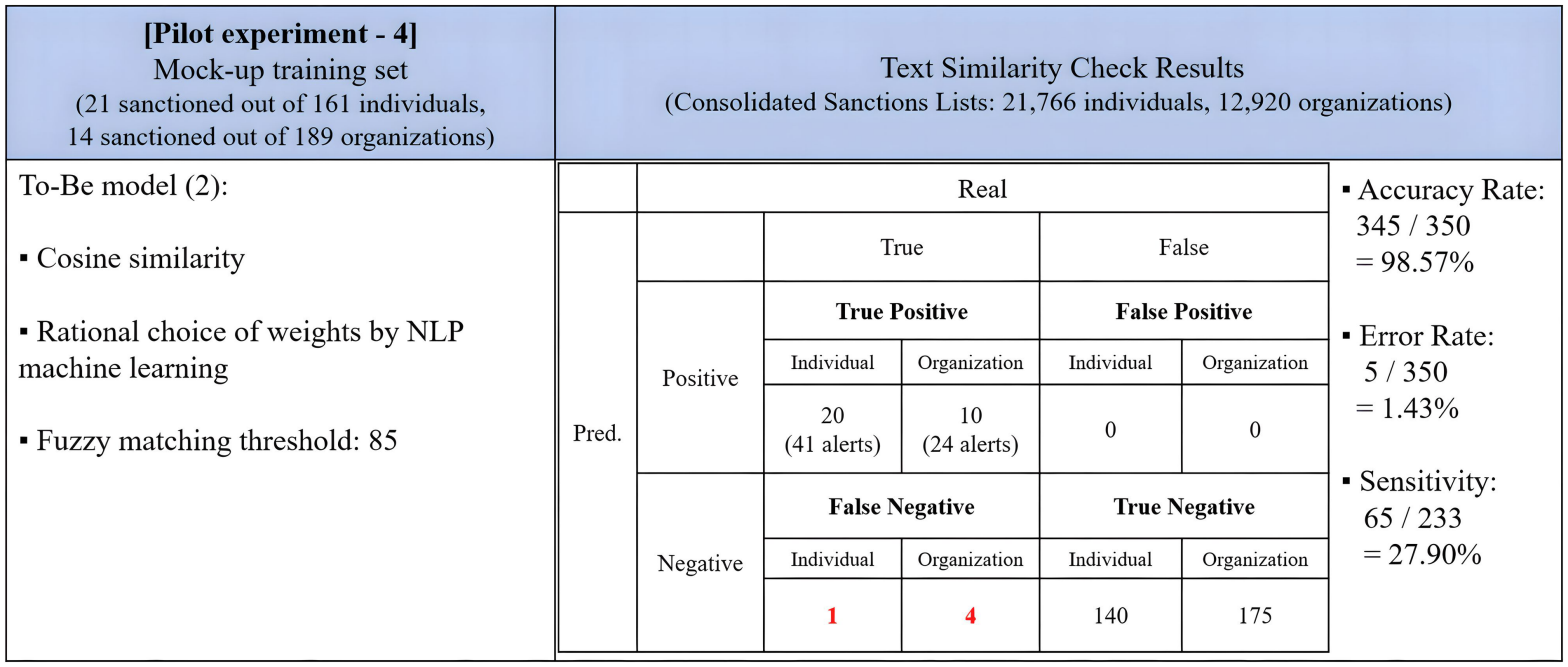
Results of the pilot experiment of NLP adoption on the cosine similarity algorithm.

In an additional step, the efficiency of the cosine similarity algorithm was examined as a text similarity check method. Cosine similarity is particularly appealing for its swift and cost-effective computation (Faruqui et al., 2016) and demonstrates a high level of accuracy. Its performance was presented in Figure 6. The same training set was used to test the program, and the fuzzy matching threshold was set at 85. NLP allocated weights to each data type, similar to the prior model, through its automated machine learning process. The execution time for this version was 14 h and 12 min, the longest among the three versions as previously mentioned.

Figure 6 shows that the text similarity check with cosine similarity yielded highly accurate results but exhibited low sensitivity. The program triggered a total of 66 alerts, the fewest among the As-Is and To-Be versions based on Levenshtein distance. It correctly identified 20 individuals and 10 organizations as true positives but failed to detect a true positive individual and four true positive organizations. Its sensitivity stood at 27.90, 53.65%-points lower than the To-Be version based on Levenshtein distance. In this context, it was challenging to consider the program as a suitable sanction screening engine, as it missed true positives while producing zero false positives, rendering it insensitive to spelling variations or keyword errors.

At this stage, each of the three different programs exhibited distinctive pros and cons for use as a sanction screening program. The primary criteria for assessing their performance as a sanction screening tool are: (1) the number of false negatives and (2) the number of false positives. Sensitivity is an additional but complementary criterion for evaluating accuracy, as it can be improved through program adjustments. Based on the results, the To-Be model based on Levenshtein distance with NLP adoption best met the first criterion. This model is the most conservative, generating the most alerts, thus reducing the program’s accuracy rate and increasing its error rate. However, it exceled in capturing most of the relevant keywords of sanctioned entities with a high sensitivity in alert generation. This highlighted the need to identify the optimal threshold for the model to minimize false positive hits.

On the other hand, the To-Be model based on cosine similarity exceled in meeting the second criterion by generating no false positives, unlike the other models, which incorrectly categorized most true negatives as positives. However, its high accuracy was attributed to the low number of alerts generated, as the program’s extended execution time allowed NLP to learn and match very precisely similar keywords. This “selecting only the finest” behavior was less desirable for a sanction screening program, where the primary goal is to identify suspicious entities in advance. Despite its high accuracy, this model missed five true positives.

Table 5 presents a comparative analysis of the strengths and weaknesses of these programs and assigns a scorecard based on four criteria. The performance of each model was evaluated, with three points awarded for the finest model, two points for the middle-level, and one point for the poorest model.

**Table 5:** Strengths and weaknesses of the programs designed.

Version of the demo sanction screening program	As-Is Levenshtein + adjusted weights	To-Be Levenshtein + weights by NLP	To-Be Cosine similarity + weights by NLP
*Pros*	User can adjust the details	Conversative, catches out all true positives	The lowest number of false positives High accuracy
*Cons*	5 false negatives Too much false positives Long code execution time	The largest number of false positives	5 false negatives Low sensitivity Dangerous to adopt since it is not conservative Long code execution time
Strengths and weaknesses analysis			
*Number of false negatives*	1.5 points	3 points	1.5 points
*Number of false positives*	2 points	1 point	3 points
*Accuracy rate*	2 points	1 point	3 points
*Sensitivity*	2 points	3 points	1 point
*Total score*	7.5 points	8 points	8.5 points

While the To-Be model based on cosine similarity scored highest in the strengths and weaknesses analysis, it was not selected for the main experiment due to its vulnerability to missing true positives. The To-Be model based on Levenshtein distance was employed for further experimentation.

To identify the optimal threshold, additional tests were conducted by adjusting the fuzzy matching threshold. This time, a threshold of 90 was applied to investigate its impact. The reason for selecting a higher threshold was to assess whether the To-Be Levenshtein distance program with NLP adoption could reduce false positive cases while still capturing all true positives. Simultaneously, the As-Is model was tested under the same setting, allowing for a comparison of the two models’ performances. The results of these additional experiments are described in Table 6.

**Table 6:** Performance overview of As-Is and To-Be models under the fuzzy matching thresholds of 85 and 90.

Fuzzy matching threshold	Assessment criteria	As-Is Levenshtein with adjusted weights	To-Be Levenshtein with weights by NLP
*85*	Accuracy rate/error rate	56.00%/44.00%	39.43%/60.57%
	Number of false negatives	5	0
	Number of false positives	149	212
	Sensitivity	51.52%	81.55%
*90*	Accuracy rate/error rate	78.00%/22.0%	57.14%/42.86%
	Number of false negatives	5	1
	Number of false positives	72	149
	Sensitivity	35.50%	63.59%

Under the fuzzy matching score threshold of 85, the As-Is version achieved an accuracy of 56.00%, while the To-Be version reached an accuracy of 39.43%. Both accuracy rates increased when the threshold was raised to 90. The As-Is version saw a 22%-point improvement, a 39.29% increase from before, and the To-Be version experienced a 17.71%-point rise, marking a 44.92% improvement.

Regarding the number of false negatives, the As-Is version triggered five false negatives under both settings of fuzzy matching score thresholds of 85 and 90. The sensitivity stood at 35.50%, with the As-Is version generating 82 true positive alerts out of 231 actual positive data. In contrast, the To-Be version produced no false negatives under the threshold of 85 and just one false negative under the threshold of 90. This single false negative occurred because “Roshan Shirkat” from the EU/UN/Dutch Government’s sanctions lists received an 89.79 name match score, falling below the threshold of 90. The sensitivity was 63.95%, calculated from 149 true positive matches out of 233 true positive matches.

To extract a moderate effect of the fuzzy matching threshold, a median value of 87.5 was applied in the main experiment.

### 3.4 Measurement

In this research, we delve into the crucial aspects of accuracy and sensitivity in the context of sanction screening. These two variables are at the core of our quantitative evaluation. We emphasize the significance of avoiding false negatives and introduce the “sensitivity” criterion to address this concern. Furthermore, we shed light on the intricate relationship between real positives, true positives, and sensitivity through an illustrative example.

#### 3.4.1 Accuracy

In this research, we evaluated two key variables quantitatively: the accuracy of the text similarity check and the accuracy of the sanction screening program. These variables were assessed using the same methodology, involving the calculation of accuracy rates and error rates. To illustrate, if the input data is a true negative, but the program erroneously generates alerts for two unrelated sanctioned entities, these false positive alerts will be counted as 2. Conversely, when the input data is an actual sanctioned party, and the program correctly identifies it, a true positive hit is registered as 1. The cumulative count of both false positives and true positives were analyzed across all datasets.

The accuracy rate of the sanction screening program was determined as follows:

*Accuracy rate* = (Total number of correct assessments)/(Total number of assessments) = (True positive + True negative)/(True positive + False positive + True negative + False negative)

*Error rate* = (Total number of incorrect assessments)/(Total number of assessments) = (False positive + False negative)/(True positive + False positive + True negative + False negative)

It is important to note that the error rate encompassed both false positives and false negatives. However, given the critical importance of avoiding false negatives in sanction screening, we introduced the “sensitivity” criterion to account for this.

#### 3.4.2 Sensitivity

Sensitivity, akin to its use in medical tests, quantified the probability of correctly identifying a person with a certain condition (Zhu et al., 2010):

*Sensitivity* = (Number of true positive alerts)/(Total number of actual positive cases) = (True positive)/(True positive + False negative)

Sensitivity provided insight into the program’s ability to detect criminals and terrorists effectively, aiming for a 100% detection rate.

To assess the sensitivity of the sanction screening results, it is crucial to detect a.k.a. names. Ideally, sanctioned entities should be alerted with all their names to prevent any financial transactions under their aliases. This paper aims to identify the optimal settings through designed experiments to achieve a high accuracy rate and sensitivity.

While sensitivity is a critical metric for evaluating the program’s performance, it is considered a supporting criterion in this paper. This is because the system can be enhanced to alert all data associated with identical information of DOB and POB when an alert is required by an entity with the same background information.

#### 3.4.3 Real positives, true positives and sensitivity

In the mock-up data set, 30 individuals and 20 organizations were randomly selected from the sanctions lists, with their official names included. This implied that each entity may have one or more alias (a.k.a.) names listed on the sanctions. If the program generated at least one name match alert for a sanctioned entity, it was classified as a true positive. A true positive was a genuine positive case that the program successfully identified, and it encompassed both true positives and uncaught true positives. Therefore, a true positive was quantifiable when the program triggered an alert, corresponding to the data that necessitates an alert. On the other hand, true positive cases required validation against the sanctions lists, as the program may not visualize them.

**Figure 7:**
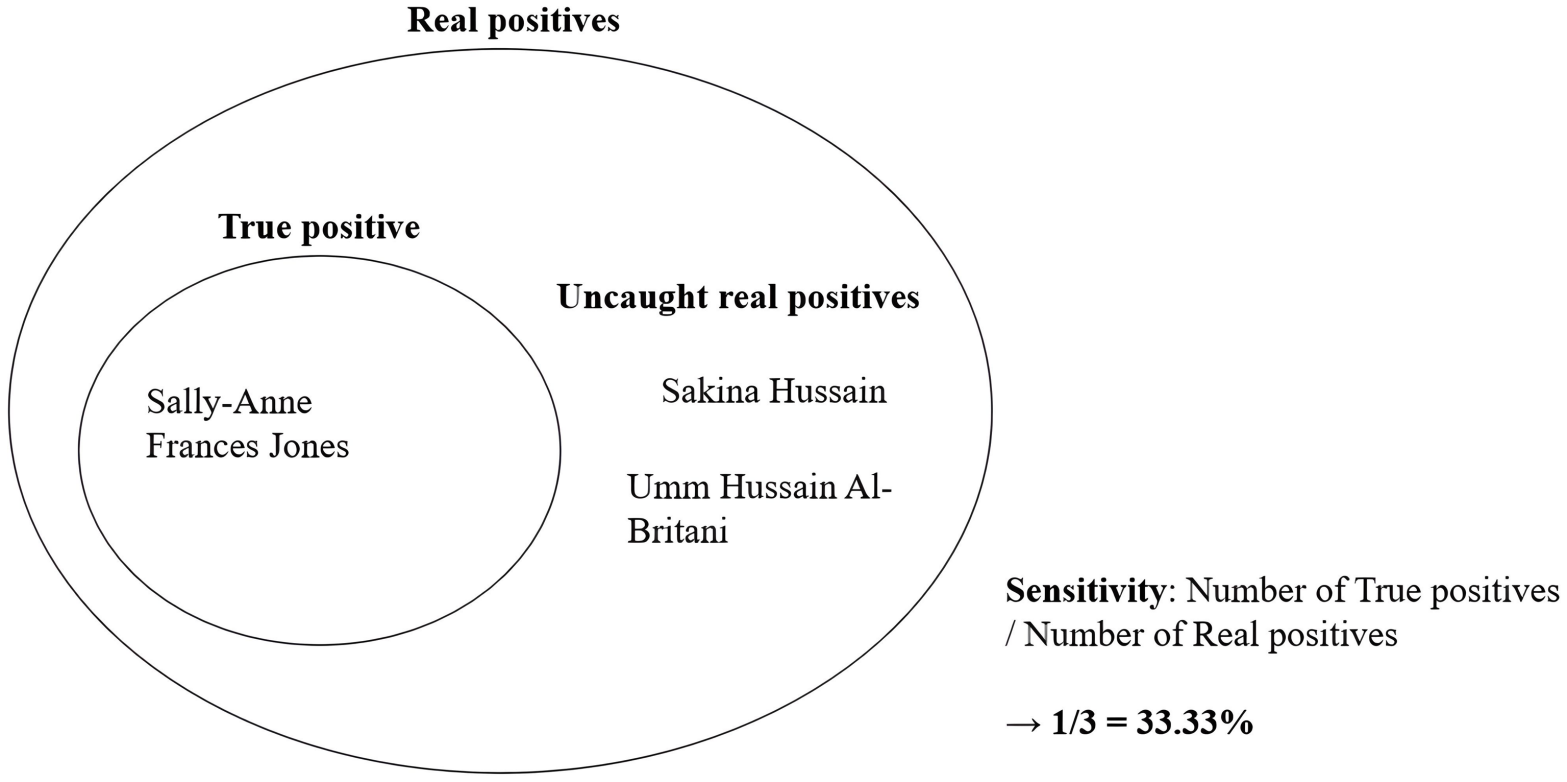
Concepts of real positive, true positive, and sensitivity.

What caused the discrepancy between the number of real positives and true positives? At times, official names and their aliases could appear substantially different. For instance, consider the case of “Sally-Anne Frances Jones,” who was born on 17th November 1968 in Greenwich, United Kingdom and was sanctioned by OFAC and EU due to her involvement as a recruiter and propagandist for the Islamic State of Iraq and the Levant. During her terrorist activities, she adopted aliases such as “Sakina Hussain” and “Umm Hussain Al-Britani.” In such instances, when official names and aliases seem unrelated, it became challenging for the program to match the aliases with the input of the official name. Figure 7 below illustrates the relationship between real positives, true positives, and sensitivity using this example.

### 3.5 Data collection

Two primary datasets were required for the experiment. The first dataset consisted of sanctions lists published by OFAC, EU, UN, and the Dutch government. This dataset served as a benchmark for evaluating the program’s alert generation. The program generated an alert when new input data matches any entry on the sanctions list. This initial dataset was assembled from the official websites of these organizations and consolidated into a unified dataset. Table 7 provides a summary of the data collection process for the sanctions lists as a benchmark dataset.

**Table 7:** Description of sanctions lists data.

	OFAC sanction list	EU/UN/Dutch sanction lists
*Individual*	13,682	8,084
*Organization*	11,495	1,425
*Total*	25,177	9,509

The second dataset was the mock-up dataset used to test the performance of the demonstration version of the sanction screening program. This dataset comprised random names, DOB, POB, and exact names of entities found on the sanctions lists. Since actual customer data from a bank was confidential and inaccessible, we created this dataset ourselves. Its purpose was to assess the accuracy of the screening results. High accuracy implied that the model was suitable for real-world data. The construction of the mock-up dataset involved five main stages, as outlined in Table 8.

**Table 8:** Five-stage process of the mock-up data construction.

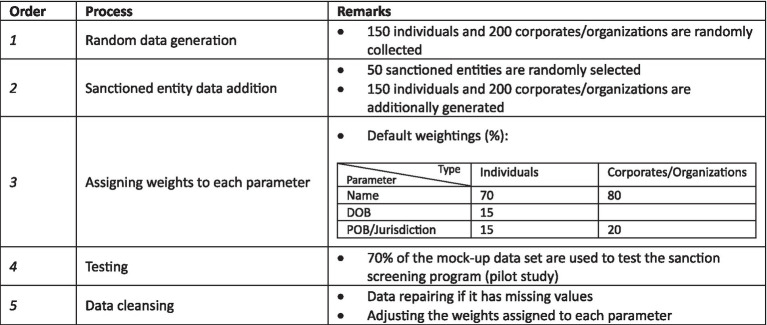

The first stage involved collecting common and realistic human names by web searching. We gathered 100 widely used male and female names in the Netherlands, along with Dutch surnames with a significant population, to create a list of “innocent” individuals’ names.

An additional 50 random names were generated using the “Random Name Generator” tool, which allowed for customization in terms of gender and other parameters. These names were adjusted to follow a specific format.

The next 200 random names and jurisdictions of companies and institutions were sourced from Forbes Global 2000 and the List of International Organizations in the Netherlands. Care was taken to ensure that names selected in this process did not overlap with sanctioned entities.

In the second stage, 50 random data selections were made from the sanctions lists of OFAC, EU, UN, and the Dutch government. These selections included 30 individuals and 20 organizations. They must be correctly alerted by the sanction program as true positives since they originated from the benchmark dataset.

To create an additional 100 data entries, 50 individuals and 50 organizations were manually crafted to closely resemble the information of the 50 sanctioned entities. This was done to observe false positive hits, which directly relate to accuracy.

As described in Table 9, the mock-up dataset comprised 500 customer data entries, which included 200 non-sanctioned individuals, 250 non-sanctioned organizations, and 50 sanctioned entities. Given the innovative nature of our study on text-matching techniques in banking, determining the optimal sample size posed a unique challenge due to the lack of direct references in the literature. To overcome this, we relied on expert consultations and practical considerations, which together provided a sound basis for our sample size decisions.

**Table 9:** Structure of the mock-up dataset.

Data type	Number of data	Configuration
Individuals	200	150 are randomly generated. 50 are manually adjusted to be similar to the sanctioned parties.
Organizations	250	200 are randomly collected 50 are manually adjusted to be similar to the sanctioned parties
Sanctioned entities	50	Randomly selected from the sanction lists
Total	500	

The third stage involved assigning weights to each parameter, determining the importance of each parameter in the text-matching process. For instance, the name parameter might receive 70% of the total importance, while DOB and POB each received 15%. POB was further divided into country and city, with each subcategory assigned 7.5% of the total weights. The assigned weights influenced the possibility of alerts, with higher weights indicating greater importance. The order of the text similarity check also followed the assigned weights.

The fourth stage involved testing the program with a subset of the mock-up data, using 350 partial data entries to train the model as part of the pilot study mentioned in Section 3.3.

The fifth stage, data cleansing, involved repairing data with missing values based on the results of the sanction screening program. In actual banking practice, bankers contact customers to obtain missing information for sanction screening. Additionally, the weights assigned to each parameter may be adjusted based on feedback to improve the screening program’s performance.

## 4 Research findings

This section presents the outcomes of the conducted experiment. Detailed progress and findings from each phase of the experiment will be expounded upon in the forthcoming paragraphs. Furthermore, we will provide explanations aimed at facilitating an understanding of the implications and insights gleaned, ultimately guiding the conclusion for each hypothesis.

### 4.1 Phase 1: As-Is version

Phase 1 of the experiment aimed to implement the existing As-Is sanction screening model and assess its performance. Based on the adjusted settings detailed in section 3.3, a Levenshtein distance text similarity check was conducted. The experiment employed a mock-up training set consisting of data for 230 individuals and 270 organizations. Among these, 30 individuals and 20 organizations were intentionally made identical to entries on the sanctions lists to evaluate whether the demo sanction screening program correctly filtered them.

**Figure 8:**
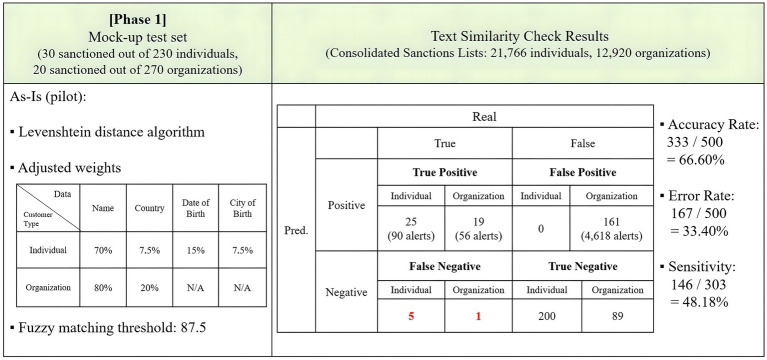
Results of the As-Is model.

The first test in phase 1 involved adjusted fuzzy matching weights and a threshold of 87.5. The results are displayed in Figure 8. The As-Is model achieved an accuracy rate of 66.60% and an error rate of 33.40% in text matching. It correctly predicted 44 true positives and 289 true negatives. However, it made erroneous predictions by labeling six true positives as negatives and generated 161 false positives, resulting in 4,618 hits. The sensitivity stood at 48.18%, as the program identified 146 distinct true positive names out of the 303 true positives on the benchmark sanctions lists.

The occurrence of false negatives, especially six cases involving true positives misclassified as negatives, is a critical issue. Given the severe implications of false negatives, including potential legal and regulatory consequences for banks, this result underscores the need for more effective screening to minimize these critical errors, prompting the next phases of the experiment to explore whether NLP adoption can mitigate these issues.

### 4.2 Phase 2: To-Be version

The second phase of the experiment implemented the To-Be model with fuzzy matching weights calculated by NLP. The test involved 500 identical data inputs subjected to a fuzzy matching threshold of 87.5.

**Figure 9:**
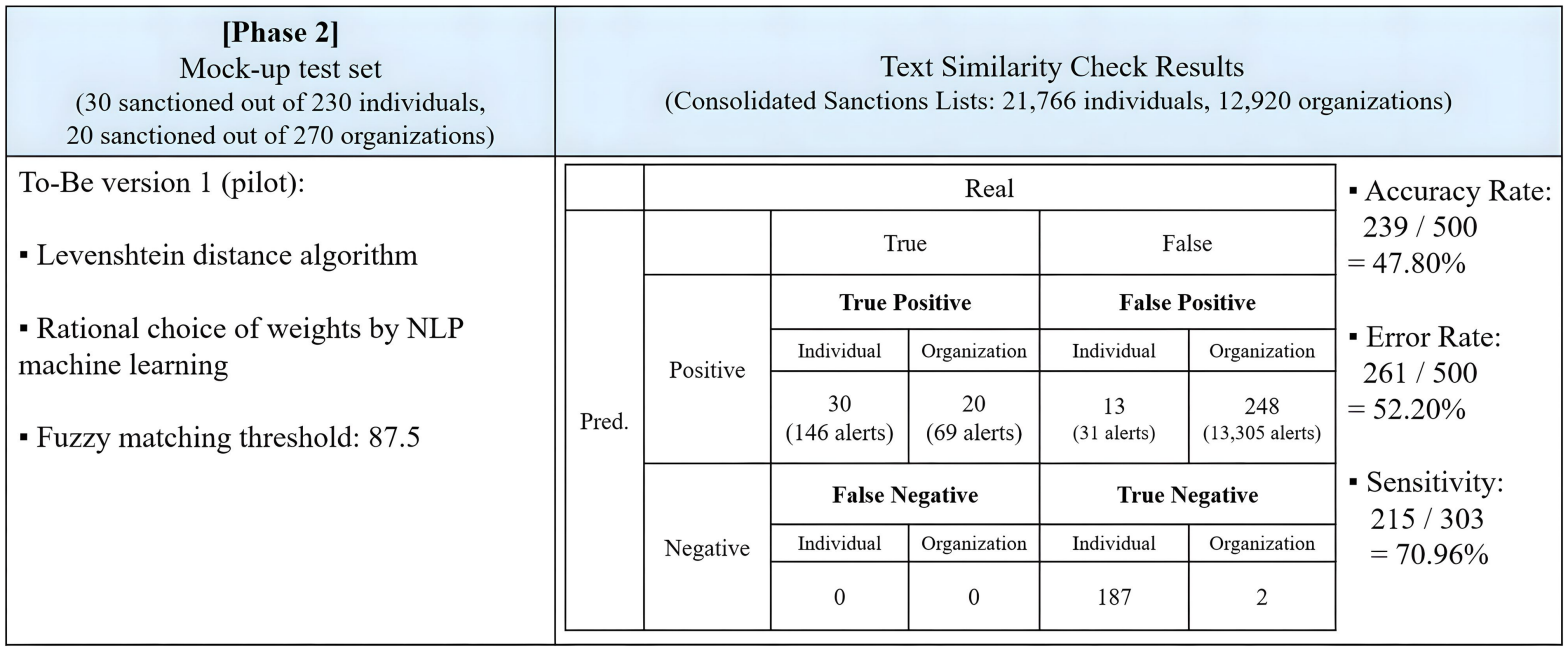
Results of the To-Be model with NLP fuzzy matching weights.

Figure 9 summarizes the results of the test. The To-Be model with NLP-derived fuzzy matching weights effectively identified all true positives as true positives but had a lower accuracy rate than the As-Is model. It correctly identified all 50 sanctioned entities with 215 true positive hits out of 303 true positives, achieving a sensitivity of 70.96%, 22.78 percentage points higher than the As-Is model. However, it generated numerous false positive alerts, wrongly predicting 261 out of 450 innocent entities as positive, resulting in 13,336 false positive alerts. The accuracy rate stood at 47.80%, and the error rate was 52.20%.

In the second test of phase 2, the experiment applied NLP to fuzzy matching weighting schemes. The To-Be model in phase 2 successfully identified all true positives, totaling 50, with no false negatives. Sensitivity remained constant at 70.96%. However, the number of false positives increased to 276, leading to a total of 31,581 false positive alerts. Compared to the first test in Figure 8, accuracy decreased by 3.00 percentage points.

A comparison between the complete As-Is model in Figure 8 and the To-Be version in Figure 9 shows that the accuracy rate decreased from 66.60 to 47.80%, while sensitivity increased from 48.18 to 70.96%. The number of false negatives reduced from 5 to 0 for individuals and from 1 to 0 for organizations. In contrast, the To-Be version excelled in detecting true positives, increasing from 25 to 30 for individuals and from 19 to 20 for organizations. However, the To-Be model fell short in accurately matching the input with the sanctions lists, resulting in lower overall accuracy.

Concluding that the adoption of NLP on the fuzzy matching weights will definitively increase the accuracy of text similarity checks at this point is a challenge. The To-Be model, with NLP adoption, exceled in detecting true positives but experienced a decrease in overall prediction accuracy. While the To-Be version prioritized the primary goal of the sanction screening program, making it more valuable, it was essential for the program’s overall accuracy to surpass that of the As-Is version. The need to reduce false positives generated by the To-Be version becomes evident.

The findings suggest that when NLP is incorporated, the program’s overall accuracy decreases, but sensitivity and accuracy in detecting true positives improve. This indicates that NLP adoption in fuzzy matching weighting schemes tends to make the program more conservative in generating alerts, resulting in increased sensitivity but decreased accuracy.

Given the severe consequences of false negatives, including potential legal actions and regulatory fines, the improved sensitivity and reduction of false negatives in the To-Be version highlight a significant advancement. Although this comes at the cost of increased false positives, the primary goal of the sanction screening program is to prevent illicit transactions and ensure compliance. Therefore, the trade-off is justified as the reduction in false negatives represents a substantial improvement in achieving regulatory compliance and preventing financial crimes.

In short, the To-Be version with NLP-derived fuzzy matching weights demonstrated a significant improvement in sensitivity, reducing the incidence of false negatives, which are the most critical errors in sanction screening. Although this came at the expense of an increased number of false positives, the trade-off is acceptable given the severe implications of failing to detect sanctioned entities. The improved sensitivity aligns with the primary goal of the sanction screening process, reinforcing the importance of prioritizing the reduction of false negatives to enhance compliance and security in financial transactions.

## 5 Discussion

The domain of banking sanction screening procedures is evolving toward increased automation and technological advancement to enhance cost-efficiency and time-saving measures (Turki et al., 2020; Achanta, 2018). In this context, Machine Learning and NLP have gained recognition as technologies of interest for financial institutions. Alkhalili et al. (2021) have suggested the effectiveness of adopting Machine Learning algorithms for watchlist filtering in transaction monitoring, leading to higher accuracy rates. However, these AI technologies face challenges in gaining acceptance due to their novelty and unverified performance in practical applications (Chartis Research Staff, 2019).

Amid the current trend of exploring new technologies to enhance sanction screening accuracy, this paper investigates the potential of NLP implementation in the sanction screening process. To connect these objectives, the paper focuses on the technical aspects of sanction screening, with a specific emphasis on the accuracy of the text similarity check. Building on previous findings regarding the efficacy of NLP in improving fuzzy matching (Bhasuran et al., 2016), this paper introduces NLP to the fuzzy matching weights to assess its impact on sanction screening. The experiment results yield the following three key takeaways for discussion.

### 5.1 The influence of NLP adoption on the sanction screening program

**Figure 10:**
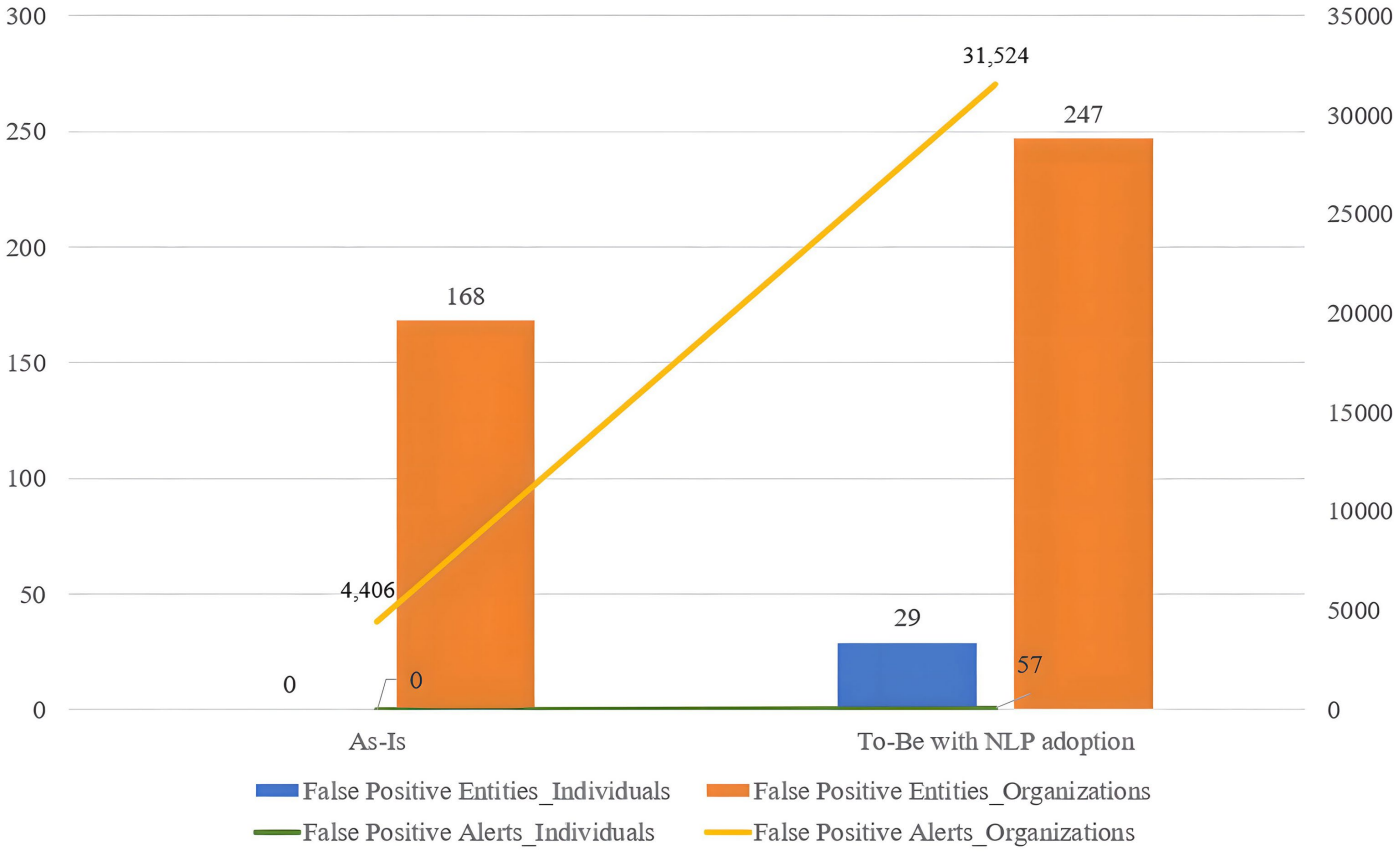
The number of false positives and alerts occurrence in individual/organization sanction screening.

At any phase of the experiment, the program generated fewer alerts for individual screening compared to organization screening. Figure 10 illustrates the difference in the number of false positive entities and alerts between the two customer types. The blue bar and green line represent the number of false positive individuals and alerts in each phase, while the orange bar and yellow line represent the number of false positive organizations and alerts in each phase. Despite the larger number of organization test sets (270) compared to individuals (230), the program triggered few false positives for individuals but wrongly predicts the majority of innocent organizations as positive.

This disparity arises from the amount of information available for processing in the sanction screening process. For individuals, four types of information – name, DOB, city of birth, and country of birth—are collected, while organizations provided only two types of information: name and country of operation. The quality of data from both customers and sanctions lists played a significant role in sanction screening accuracy. The availability of richer information helps specify the identity of the inputted customer, reducing unnecessary false positives and alerts by enhancing the accuracy of name matching. Therefore, the collection and management of an adequate quantity of high-quality data are crucial factors influencing sanction screening accuracy.

While sanction screening for individuals performed well due to the volume of available data, it resulted in more false negative cases, as observed in the As-Is model (five for individuals and one for organizations), compared to the To-Be model (zero for both individuals and organizations). This indicates that sanction screening with detailed information generates fewer false positives but also fewer correct predictions.

Given the critical importance of avoiding false negatives, the improved sensitivity in the To-Be model, despite generating more false positives, represents a significant advancement. The reduction of false negatives, which pose the greatest risk in terms of regulatory compliance and financial crime prevention, justifies the increased number of false positives. The trade-off is necessary to prioritize the detection of all potential threats.

### 5.2 The gray area in assessing the sanction screening program’s performance

**Figure 11:**
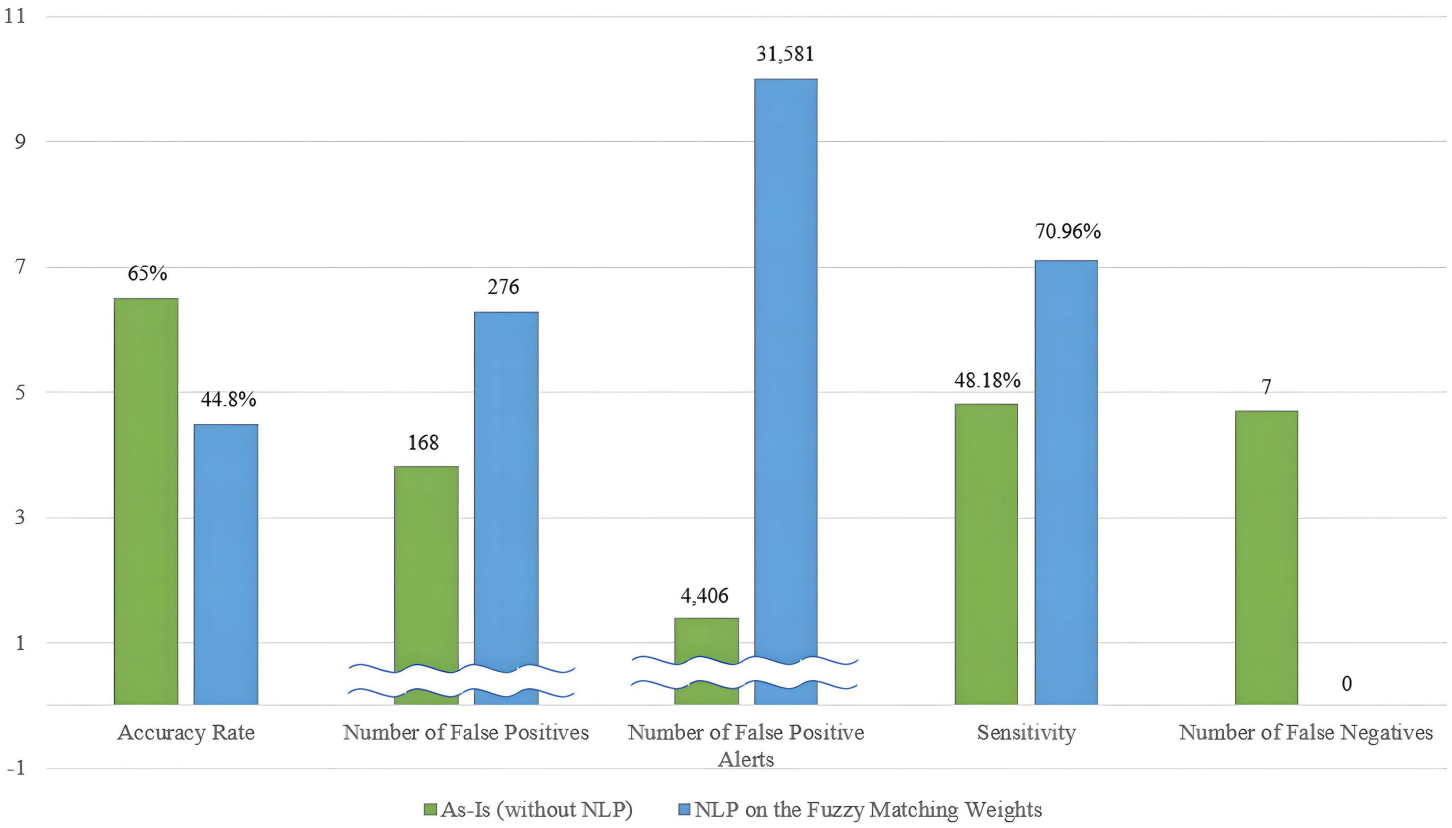
Tradeoffs between general accuracy and sensitivity in sanction screening.

A significant challenge is the difficulty in definitively concluding which sanction screening model is superior. While this paper aims to enhance the sanction screening program’s accuracy and sensitivity, it reveals a trade-off between these two objectives. The As-Is program, which relies on the Levenshtein distance algorithm and weighting schemes, demonstrates a tendency toward achieving a high overall accuracy rate but has limitations in identifying true positive entities, leading to a few false negative cases and low sensitivity. In contrast, the To-Be model, based on the Levenshtein distance algorithm but with weightings influenced by NLP during the process, excels in identifying true positive matches with high sensitivity but exhibits relatively lower accuracy in the overall context. Figure 11 visually illustrates this trade-off between the two criteria.

This trade-off suggests that achieving both goals simultaneously has limitations. Given the inverse relationship between the two distinct objectives, stakeholders must carefully consider the program’s direction based on their specific use cases. According to the theory of Value Tradeoff in normative decision-making, the three main sequences to be followed are identifying the crucial problem, justifying the procedure and its contribution, and exploring potential solutions (Hadari, 1988).

The issue addressed in this paper is the improvement of the sanction screening program’s accuracy in terms of both overall accuracy and sensitivity. In general, the priority often aligns with achieving zero false negatives, which closely relates to sensitivity. However, different banks face unique constraint problems, such as issues related to the quality of sanction prevention or constraints in terms of time and labor. Therefore, the choice of which requirement to prioritize—achieving general accuracy in text matching or detecting hidden positives based on the use case (Deloitte, 2020)—becomes crucial.

For banks struggling with a significant workload in managing false positive alerts, sticking with the As-Is version of the sanction screening program is advisable. On the other hand, if a bank seeks to enhance financial crime detection, adopting an NLP system alongside the existing sanction screening program may be preferable.

The key takeaway here is the recognition of the existence of gray areas in evaluating the sanction screening program’s performance. Defining the “better” model becomes challenging when accuracy and sensitivity are in conflict. This paper prioritizes the requirement of eliminating false negatives, leading to the conclusion that the sanction screening program with NLP adoption represents an improved solution, as demonstrated in the experiments. However, this conclusion may vary if the priority requirements change. For instance, if accuracy and sensitivity are equally important, it becomes challenging to argue that the NLP-enhanced sanction screening program is superior to the original version.

To evaluate the quality of software development, substantial empirical evidence from literature and real-life decision makers must precede (Biffl et al., 2008). Given that research on the collaboration of NLP and sanction screening programs is in its early stages, further studies are expected to provide valuable insights for determining the preferred criteria in the future.

### 5.3 Key factors affecting the accuracy of the sanction screening program

The experiment highlights the significance of data quality and specificity as crucial factors influencing the accuracy of sanction screening. As Kokemüller (2010) underscores the risks associated with poor data quality that can impact sanction screening accuracy, the contrasting results between individual data with detailed information and organization data with less precision underscore the pivotal role of data quality in sanction screening.

In the experiment, the test set was categorized into two customer types: individuals and organizations. Individual data was accompanied by four specific information categories, including name, DOB, city of birth, and country of birth, all of which were employed in text matching. In contrast, organization data was limited to two categories, name and country of operation. Consequently, the program exhibited higher accuracy in individual sanction screening, generating relatively fewer false positives.

However, in the case of organization sanction screening, the program became less efficient, resulting in a significant number of false positive alerts in both the As-Is and To-Be models. Therefore, the collection of comprehensive and accurate data, along with diligent data quality monitoring, is essential for achieving highly reliable sanction screening results.

## 6 Conclusion

This section serves as a concise overview of the primary discoveries and addresses the core research question posed in this paper. Following this, the section will underscore the academic and managerial contributions arising from this research. To conclude, we will outline the limitations inherent in this study and offer recommendations for future research endeavors.

### 6.1 Key findings

As global reported cases of financial crimes continue to rise (Financial Conduct Authority, 2021), and associated costs surge (Refinitiv, 2018), the banking sector faces an escalating need for precise sanction screening to safeguard both the financial industry and its own integrity. The core mission of a sanction screening program is to identify and alert potential positive matches among sanctioned entities before any transaction occurs. A false negative prediction is a critical failure, making the accuracy of results a paramount concern. Remarkably, over 90% of alerts generated by current sanction screening programs ultimately turn out to be false positives (Goethals and Decraemere, 2020; Dean, 2022), underscoring the critical challenge of enhancing result accuracy.

The introduction of NLP into the sanction screening program, specifically via modifications to fuzzy matching weights, has a mixed impact on the accuracy of text similarity checks. While the overall accuracy rate diminishes, sensitivity improves significantly, resulting in a reduced incidence of false negatives.

The primary goal of any sanction screening program is to prevent false negatives, as they pose the most severe risks, including regulatory penalties and facilitating financial crimes. The findings of this study highlight that the To-Be model with NLP-derived fuzzy matching weights significantly improves sensitivity, thereby reducing false negatives to zero. Although this improvement comes at the cost of increased false positives, the trade-off is justified given the higher stakes associated with false negatives. This consistent emphasis on reducing false negatives aligns with the critical importance of ensuring compliance and preventing illicit activities.

### 6.2 Academic contributions

This research contributes to the academic domain in several significant ways, setting the stage for future studies. Firstly, it brings the topic of sanction screening programs into the academic sphere, which has seen limited exploration. As sanction screening technology originates from practical applications within the financial industry, there’s a dearth of academic reviews published in scholarly journals. Typically, this topic has been discussed in white papers or reports by private risk solution consultancies, with limited accessibility. This paper introduces this niche topic to academia, bridging banking practice and theoretical review, with the expectation of fostering scholarly interest in banking sanction screening solutions.

Secondly, this research suggests new potential for incorporating NLP into FinTech, expanding knowledge of effective sanction screening solutions and NLP technology applications. Historically, the banking sector has been hesitant to adopt NLP due to concerns about unproven performance and related risks (Chartis Research Staff, 2019). By evaluating the validity of implementing NLP in sanction screening systems to enhance screening result accuracy, this paper aims to bridge the gap between existing system outcomes and theoretical approaches, providing empirically verified information on NLP within an academic context.

Furthermore, this paper makes a noteworthy contribution to the realm of Business Information Management. It accomplishes this by establishing a critical connection between real-world business practices and innovative data management strategies. In response to the challenges presented by the existing sanction screening system, the paper not only identifies these challenges but also provides concrete solutions. These proposed remedies offer invaluable insights into data management and the application of text similarity technologies, enriching the academic discourse within this field. This integration of practical business approaches with academic research serves to enhance and advance our understanding of this dynamic and ever-evolving domain.

### 6.3 Managerial implications

This research, offering insights into the application of NLP in FinTech, provides valuable managerial implications to stakeholders in the banking industry.

Firstly, financial institutions can use this research to improve their banking sanction compliance practices. Given that sanction screening aims to prevent financial institutions from engaging in illegal financial transactions and money laundering, this research sheds light on better management of customer data and sanctions lists. Financial institutions can establish robust plans to develop internal sanction screening tools tailored to their specific requirements, prioritizing either high sensitivity or high general accuracy based on their needs.

Secondly, solution vendors and internal IT officers within banks can leverage the insights provided by this research to enhance their programs. As the accuracy of sanction screening programs is a pressing concern, this research offers practical solutions to address these accuracy issues. It can serve as a starting point for more advanced and professional research, with results that can be directly applied in practice.

Thirdly, the compliance department staff will benefit from the reduction in manual sanction screening workload. Although this paper does not provide a solution that eliminates both false positives and false negatives, reducing either of these aspects eases the burden of manual screening. This leads to time and cost savings in manual checks, enabling compliance staff to allocate their time more efficiently to other human-centered tasks.

Fourthly, technology solution consulting firms can utilize the findings of this research in their future consultancy projects. This research serves as a reference source to guide their bank clients in adopting effective and validated technological solutions that are both novel and practical.

Ultimately, this research contributes to the broader goal of combatting financial crime and upholding sanctions regulations, aligning with societal expectations to protect the security of society.

### 6.4 Limitations and future research

This section addresses both the limitations of our research and proposes future directions to build upon these limitations and strengthen the empirical foundation of sanction screening technologies.

One limitation of this study is the use of a demonstration version of the sanction screening program, which replicates only the fundamental features of real-world systems employed by banks. While this simplified model offers valuable insights, it does not fully capture the complexity of actual systems developed by solution vendors who use sophisticated techniques to ensure both accuracy and efficiency in processing vast amounts of data (Oracle, 2022). Additionally, real-world banking data often presents significant class imbalances between sanctioned and non-sanctioned entities, typically skewing toward a higher volume of non-sanctioned organizations. This imbalance can affect the generalizability of our findings, as the results from NLP-based fuzzy matching may differ in real-world systems. Future research should address this limitation by developing a more sophisticated version of the demo program and collaborating with solution vendors such as Oracle and LexisNexis to ensure the findings align more closely with the complexities of operational systems and real-world data challenges.

Another limitation lies in the controlled nature of the dataset used in this research, which contained four categories of clean and complete information. In real-world banking environments, however, data is often incomplete or inconsistent, and sanction screening frequently relies heavily on name matching due to limited access to additional identifying information, such as dates or places of birth. Future research should investigate the impact of data quality on sanction screening outcomes and explore which data types and amounts are most critical for achieving accurate results. Such research could provide valuable insights for improving Know Your Customer (KYC) processes and enhancing data management practices related to sanctions.

Looking ahead, there are several promising avenues for future research to expand on the current study. Developing more sophisticated sanction screening programs that better replicate real-world conditions, particularly by addressing the class imbalances between sanctioned and non-sanctioned entities, could significantly improve model performance. Techniques such as oversampling or advanced Machine Learning methods may offer viable solutions for handling unbalanced datasets. Additionally, research into how emerging technologies like Robotic Process Automation (RPA) and Quantum Computing could be integrated into the sanction screening process offers potential for further advancements. The adoption of Natural Language Processing (NLP) in sanction screening programs can also be explored beyond fuzzy matching, such as in multi-language data integration, which would enhance accuracy in global contexts.

Moreover, incorporating multivariable analysis into future studies would provide a more comprehensive understanding of the factors influencing the accuracy of sanction screening. Investigating real-time data processing techniques, enhanced fuzzy matching algorithms like the Levenshtein distance, and anomaly detection methods could also help address the evolving challenges in the field. Collaborative research between financial institutions, solution vendors, and regulatory bodies would be essential in improving data sharing practices and ensuring the continuous refinement of sanction screening technologies.

In conclusion, while our study offers significant advancements in text-matching techniques, we recognize that the challenges surrounding sanction screening remain dynamic and evolving. Our current work represents an important step forward, but future research must continue to address these limitations and explore new technologies, methods, and collaborations to fully realize the potential of enhanced sanction screening programs.

## Data Availability

The raw data supporting the conclusions of this article will be made available by the authors, without undue reservation.
